# Combined Effects of Exercise Training and Nutritional Supplementation in Cancer Patients in the Context of the COVID-19: A Perspective Study

**DOI:** 10.3389/fnut.2022.847215

**Published:** 2022-03-09

**Authors:** Mahdieh Molanouri Shamsi, Alieh Vahed, AmirHossin Ahmadi Hekmatikar, Katsuhiko Suzuki

**Affiliations:** ^1^Department of Physical Education and Sport Sciences, Faculty of Humanities, Tarbiat Modares University, Tehran, Iran; ^2^Faculty of Humanities, Tarbiat Modares University, Tehran, Iran; ^3^Faculty of Sport Sciences, Waseda University, Tokorozawa, Japan

**Keywords:** aerobic exercise training, cancer, immune response, vitamin D, coronavirus

## Abstract

The 2019 coronavirus (COVID-19) epidemic, has caused unprecedented global social and economic impacts and many deaths. Many risk factors have been identified in the progression of COVID-19 to severe and critical stages, and it is shown that the coronavirus appears more severely in people with cancer. Pro-inflammatory status and weakened immune system due to cancer-related treatments can be determinants in the immune system’s response to the coronavirus in these patients. Higher physical activity levels are associated with lower hospitalization rates and mortality in COVID-19. Also, regular exercise training can improve immune system responses, modulate inflammatory responses, and improve psychological parameters in cancer patients. The interactive effects of nutritional supplements on immune responses and anti-inflammatory status have been shown in some studies. The purpose of this perspective article was to investigate the interaction between dietary supplementation and regular physical exercise in controlling risk factors associated with coronavirus in cancer patients. In addition to appropriate dietary habits, some nutritional supplements, especially vitamin D, have been shown to improve the immune system’s response against COVID-19 and cancer. Using lifestyle strategies such as regular physical activity and intake of functional compounds as supplements can be effective in treatment outcomes, quality of life, and overall survival in cancer patients. We proposed that combining dietary supplements and exercise training in cancer patients can boost immune responses against COVID-19 and probably improve vaccine responses. Angiotensin (ANG)-(1-7) Mas receptor axis can probably activate following exercise training and vitamin D combination. And can prevent pulmonary injury, hematological alterations, and hyperinflammatory state in COVID-19.

## Introduction

Humans are exposed to different viruses; and the human body is rapidly involved in an immune response to eradicate the virus, with a pattern of detection and the production of memory cells (1). Coronavirus (COVID-19) has been observed in China since 2019 and has become an epidemic (2). The virus is caused by acute respiratory syndrome (SARS) Coronavirus 2 (SARS-CoV-2). According to the statistics published as of September 21, 2021, the number of infected people worldwide was approximately 230 million; and the number of victims was about 4.7 million, which continues so far, and 14% of patients experiences acute and severe conditions (3). Coronavirus can cause uncontrolled releases of pro-inflammatory cytokines, which leads to cytokine release syndrome (CRS) or “cytokine storm” (4). Activation of CRS can worsen acute respiratory syndrome and lead to multiple organ dysfunction (4). Evidence suggests that patients with SARS-CoV-2, who previously suffered from rheumatological immune diseases and other inflammatory diseases are more fragile to an acute respiratory syndrome caused by CRS (5). Despite global vaccinations to tackle this pandemic, we can see new infections and the prevalence of new variants. Still observing social distancing and staying at home is one of the main ways to keep the COVID-19 under control (6, 7).

In particular, cancer patients appear to be a high-risk category to experience COVID-19 with more severe symptoms, especially due to damage to immune defenses and the consequences of anti-neoplastic treatments (8). Chronic cancer-related inflammation can create an immunosuppressive tumor microenvironment to help the tumor to escape immune monitoring (9). Also, cancer is associated with over-expression of immunosuppressive cytokines, decreased proinflammatory risk signals, and increased populations of functional immunosuppressive leukocytes, which weaken the immune responses and increase the risk of infectious complications (10, 11). Therefore, these patients cannot show effective immune responses when exposed to the virus.

In addition to regular cancer treatments, appropriate dietary habits and regular physical exercise have been considered in these patients. Regular physical exercise training can induce positive changes in anxiety, depression, immune and physiological responses in cancer (12). Moreover, some dietary supplements have always been considered to boost immune responses in cancer patients (13). Higher physical activity levels are associated with lower hospitalization rates and mortality in COVID-19 (14). Positive effects of acute or chronic physical exercise were observed on innate and acquired immune responses and modulation of inflammatory status (15). Also, the effects of regular physical exercise on angiotensin-converting enzyme 2 (ACE2) as an effective agent in the pathogenesis of COVID-19 are suggested in some studies (16). In addition, the effects of physical exercise on nitric oxide and oxidative stress have been proposed as possible effective mechanisms in the prevention and recovery of the COVID-19 (17). Impaired immune responses, inflammatory status, and oxidative stress have been observed in cancer patients (18). Also, some studies showed different expressions of ACE2 in cancer patients, which makes them more susceptible to SARS-CoV-2 (19). The modulatory effects of regular physical exercise on ACE2 and its receptors may be effective in preventing severe cases of COVID-19 in cancer patients.

In addition, the positive effects of various dietary supplements such as probiotics, omega-3 fatty acids, multivitamins, or vitamin D supplements in COVID-19 are suggested in some studies (20). Considering the observed effects of dietary supplements and physical exercise in cancer and COVID-19, synergic effects of combining regular physical exercise and supplementation can improve the immune system responses in cancer patients against coronavirus. The possible effects of combining regular physical exercise and dietary supplementation in cancer and COVID-19 were discussed following a systematic search in Scopus, Web of Science, ISC, and Pub-Med databases. And the results were divided into two main topics 1) Physical Exercise in Cancer and COVID-19, 2) Nutritional Supplements in Cancer and COVID-19.

## Physical Exercise in Cancer and COVID-19

Angiotensin-converting enzyme 2 (ACE2) is one of the causes of SARS-CoV-2 entry and infection (21). It has recently been found that ACE2 receptors are also more common in cancer patients (19). Once CoV-2 entry into the target cell, the host response is determinant of the severity of the ensuing pathogenesis (22). The immune system plays a critical role in COVID-19, and in addition to the genetic profile, environmental indices can be effective on the immune responses (23).

The first immune system responses are when the COVID-19 enters the body through the innate immune system. Inflammation is the body increases when a person becomes infected with COVID-19. As seen in patients with SARS-CoV, Viral infection and proliferation in airway epithelial cells can cause high levels of virus-associated pyroptosis associated with vascular leakage (24). During inflammation, type I interferons (IFNs) are activated by the innate immune system, and the presence of type I interferons can regulate myeloid cell activation and migration (25). Rapid and appropriate activation of type I IFNs can effectively limit virus replication and reduce immune pathological damage. It also limits the hyperactive inflammatory response (26). Also, in the innate immune system, natural killer cells (NK) play a vital role in infectious diseases. NK cells decreased in patients with COVID-19 (27). Activation of NK cells lead to cytotoxic degranulation and production of inflammatory cytokines and destroys target cells (28). In addition, the adaptive immune system would activate to help innate immunity for further controlling the infection. The three critical components of the adaptive immune system against SARS-CoV-2 are B cells, CD4^+^ T cells, and CD8^+^ T cells (29). CD4^+^ T cell responses are more prominent than CD8^+^ T cell responses and play a role in controlling primary infection earlier (30). It has also been shown that the function of B cells is very important in controlling viral infections. But the critical point is that COVID-19 can disrupt the phenotype of T and B cells and reduce their function (29).

Regular exercise throughout life is effective in cardiovascular fitness and self-reported mood, anxiety, and depressive symptoms. In addition to the physiological effects of regular physical exercise, it can improve immune responses. Moderate intensity training is directly associated with a lower upper respiratory tract infection (URTI) (31). Regular physical exercise can improve innate and acquired immune system responses (32). In particular, secondary antibody responses to some traditional vaccines approved after exercise training, especially in elderly people (33).

Moreover, it is shown that physical exercise has anti-inflammatory effects, especially in chronic diseases and obesity (34). Modulation of adipose tissue macrophages, the release of anti-inflammatory cytokines after acute physical exercise, and interaction of skeletal muscle and immune system can be effective in exercise-induced anti-inflammatory effects (34). The anti-inflammatory effects of exercise and changes in various indicators of the innate immune system following acute physical exercise can improve the innate immune system responses to infections. In addition, regular physical exercise training creates effective responses in the acquired immune system especially in elderly people (35). Physical exercise has been shown to reduce mortality from illnesses such as the flu, and higher levels of physical activity have reduced the incidence of severe cases, hospitalization rates, and mortality in COVID-19 (36, 37).

Cancer-related inflammation is a prominent feature of cancer (38). Cancer-related inflammation, which in different stages of tumorigenesis, contributes to genomic instability, epigenetic modification, induction of cancer cell proliferation, strengthening cancer anti-apoptosis pathways, stimulating angiogenesis, and ultimately spreading cancer (38). In the early stages of tumor growth, cytotoxic immune cells such as NK cells and T^+^CD8 cells detect and destroy cancer cells (39). In addition, the interaction between NK cells, effective T cells, and antitumor macrophages by secreting IFN-γ and tumor necrosis factor-alpha (TNF-α) at the tumor site increases the cytotoxic ability of NK cells (39). When tumors escape primary anti-tumor immunity, they undergo different strategies that shift the balance toward immune tolerance, as they reduce the effect of inherently compatible immune cells at different levels and through different mechanisms. Tumor cells escape immune attacks using two main strategies; avoid immune detection and stimulate an immunosuppressive environment. Firstly, cancer cells may lose expression of tumor antigens on the cell surface, thus preventing detection by cytotoxic T cells. In this sense, mutations and deletions may lead to low regulation of the antigen-providing device and possibly show resistance to effective T-cell molecules such as TNF-α and IFN-γ (40). Also, agents derived from cancer cells stimulate the expression of inhibitory inspection molecules such as programmed death-ligand 1 (PD-L1), CTLA β-4, and Tregs expression by tumor-derived chemokines of the immune-resistant environment by tumor-derived chemokines (18). Overall, these strategies lead to a complex and efficient system for safe escape.

Adjuvant therapies such as physical exercise that makes changes in different aspects of the immune system in cancer may improve the efficacy of current immunotherapy. The diagnosis of cancer and the treatments that cancer patients undergo have a significant impact on their mental and physical health. Some studies showed that regular physical exercise is effective in body weight and body mass index controlling, and improving patients’ quality of life and sleep quality (41). It also seems that regular exercise through myocyte secretion, decreased inflammatory factors within the tumor, decreased tumor angiogenesis, and increased expression of factors involved in apoptosis can slow tumor growth (42). Regular exercise and physical activity can prevent the disease from recurring by affecting sex steroid hormones, metabolic hormones, inflammatory markers, cytokines and adipokines, myokines, and stress hormones (43). There are also observed effects of regular and long-term exercise on immune responses to diseases such as cancer (44). Prolonged exercise promotes anti-inflammatory effects and improves immune responses in cancer patients. In addition, recent studies have shown the positive effects of physical exercise on antitumor immunity that can affect tumor growth (45). Tumor induces physiological changes in its environment such as changes in acidity and metabolism in order to suppress antitumor immunity. Physiological responses following physical exercise can increase the infiltration of macrophages, neutrophils, NK cells, and regulatory and cytosolic T lymphocytes to the tumor microenvironment, which can be effective in tumor suppression (46).

Unfortunately, cancer patients are among the groups most at risk for severe cases of COVID-19. The psychological, physiological, and especially immunological effects of physical exercise can help these patients respond better to infection and possibly even better immune responses to the vaccine. However, the effects of exercise training have always been considered along with nutritional factors. We discussed the effects of nutritional supplements on the immune system in cancer patients to prevent severe cases of COVID-19, we also discussed the combined effects of exercise and supplementation.

## Nutritional Supplements in Cancer and COVID-19

Many studies have approved that proper nutrition and some nutritional supplements can improve immune system function and reduce infection (47, 48). Long-term use of foods and supplements rich in antioxidants such as tart cherry juice, pomegranate juice, beetroot juice, creatine, omega-3 polyunsaturated fatty acids, and vitamin D3, watermelon juice has been associated with effective immune responses (49). In addition, the effect of ginger and some of its compounds against inflammation and improving immune responses have been reported in some studies (50–52).

Among vitamins, vitamin E is one of the most effective nutrients known to modulate the function of the immune system (53). There are reports of improved immune systems in human and animal specimens by taking this supplement. Improving immune function with vitamin E is clinically important because, in addition to allergic diseases such as asthma and the flu, it also affects infectious diseases such as respiratory infections (54). Also, evidence showed that vitamin E supplementation for 30 days increased Th1 immune responses, improved lung damage, and reduced influenza infection in mice (55).

In addition, Vitamin D is a fat-soluble steroid that plays an important role in regulating calcium and phosphorus levels. Vitamin D receptors are located on immune cells, and all leukocytes can synthesize the active metabolite of vitamin D. Moreover, vitamin D can act autonomously, boosting the innate immune response and inhibiting the acquired immune response (56). Low concentrations of vitamin D have been reported to be associated with upper respiratory tract infections and allergic asthma (57). Vitamin D enhances chemotaxis, antimicrobial peptides, and macrophage differentiation. It can also inhibit the maturation of DCs, the differentiation of Th1 and Th17, and boost the functions of regulatory T cells (57). Moreover, vitamin D has been reported to play an important role in influenza virus infection (58).

The American Cancer Research Institute (AICR) has always recommended a low-fat diet, high in fruits, vegetables, and whole-grain products for cancer survivors. It also considers adequate levels of major macronutrients and various vitamins and minerals necessary to maintain health (59). During cancer treatments with chemotherapy or radiotherapy, patients often experience nausea, vomiting, diarrhea, and loss of appetite, leading to fewer food combinations and weight loss (60). Supplementation with essential vitamins and minerals may seem desirable, but it may not always. Food interactions with treatment may affect the outcome of treatment. Of particular concern here are dietary supplements with antioxidant properties, but supplements without antioxidant properties may also affect the effectiveness of cancer treatment (61). Many non-oxidant supplements are widely consumed by cancer patients, although their effects on the effectiveness of chemotherapy treatments are controversial. Vitamin D is one of the non-oxidant vitamins that is probably useful in cancer treatment (62). It has been observed that vitamin D receptor (VDR) is expressed significantly in the immune system, raising the possibility that vitamin D and similar may have immunomodulating activity (63). Cell studies state that vitamin D modulates the activity of various defense and immune cells including blood monocytes, macrophages, antigen-providing cells, and activated CD4^+^ T cells or epithelial cells (64, 65). In general, vitamin D metabolites have significant anti-neoplastic activity in clinical models. The immune system is a suitable target for the anti-neoplastic effects of vitamin D. Vitamin D receptor (VDR) is expressed in different immune cells. Vitamin D can have inhibitory effects on chronic inflammation, resulting in the proliferation of immune cells. Also, it is suggested that some types of cancer may be more sensitive to the effects of vitamin D supplementation than others (66).

Another supplement that has been considered in cancer research is selenium (67). Selenium is an essential component in several major metabolic pathways, including the antioxidant defense system and the immune system. Selenium is incorporated into 30 different selenoproteins (68). Selenoproteins play an important role in antioxidant and DNA stability and may have anti-cancer effects. Also, selenium is involved in cell proliferation and apoptotic cell death in healthy and malignant cells. Low selenium levels are associated with a high prevalence of several different types of cancer and cancer mortality (69). Also, the effects of foods and supplements such as omega 3, vitamins E and C on the immune system responses in cancer have been specifically considered and confirmed. However, due to the stage and treatments related to cancer, the use of various supplements has always been cautious. Antioxidants are not always effective during cancer treatment, and also higher doses of some supplements may cause side effects. In the following, we discussed the possible effects of some supplements in combination with exercise training as a possible modulator and the possible effects in the COVID-19.

## Discussion

Limited studies have been performed on the combined effects of dietary supplementation and regular physical exercise on the prevention and treatment of COVID-19. It seems that regular physical exercise and dietary intake of functional compounds as two main parts of a proper lifestyle that can prevent severe cases of this disease. Also, it is shown that patients with chronic disease are susceptible to severe cases of COVID-19, and a combination of exercise and proper nutrition have always been effective in reducing some chronic diseases (70). So, one of the main considerations would be lifestyle changes, including increasing physical exercise and intake of functional compounds to improve immune function and induce antiviral effects. Cancer patients as one of the chronic diseases are involved in more severe cases of COVID-19 (71). It is probable that some dietary supplements especially in high doses with anti-inflammatory and antioxidant effects can interfere with cancer treatment. Combining exercise training with dietary supplements can be effective in modulating the effects of supplements in these patients and improve immune system’s response to infectious diseases, especially COVID-19.

In the athletes, nutritional supplements such as probiotics, glutamine, a variety of vitamins and minerals, selenium, etc., have always been considered to improve the immune system responses and prevent upper respiratory system infections (72). It has also been shown that the combined effects of exercise with a proper diet can be effective in the reduction of inflammation, leukocytes adhesion, and chemotaxis capacity, and creating anti-oxidative effects in metabolic syndrome and obesity. This has been especially true for the elderly due to immunosenescence (73). Regular physical exercise and dietary intake of functional compounds can induce immune-boosting and decrease the adverse effects of age-related immune dysfunction.

Cancer survivors are often highly motivated to seek about dietary choices, physical activity, and dietary supplements to improve their treatment outcomes, quality of life, and overall survival. Many dietary supplements contain levels of antioxidants that are significantly higher than the recommended dietary intake. Research has shown that taking high doses of vitamin D or selenium supplements can improve cancer and reduce tumor volume (74). On the other hand, it has been suggested that taking high doses of supplements with antioxidant activity during chemotherapy or radiation therapy may not be wise, as antioxidants can potentially reduce cellular oxidative damage to cancer cells, which contributes to the effectiveness of these therapies (75). Recent studies have suggested the possible role of exercise training in reducing the side effects of high doses of antioxidant supplementation in breast cancer tumors. The results of studying the use of high-dose vitamin D with exercise training in women with breast cancer have shown that a combination of exercise training and a high dose of vitamin D can modulate leukocytes’ cell survival-related gene expression in breast cancer survivors (76). It is suggested that response to vitamin D supplementation in cancer modulated *via* vitamin D receptor (77). The effects of physical exercise on the increase of vitamin D receptors in various tissues have been observed in some studies (78). Also, Lithgow et al. (79) showed that moderate aerobic exercise increases T-cell vitamin D receptor expression in vitamin D-deficient men (79). Changes in vitamin D receptors in various tissues, including the immune system following physical exercise, can be used as a mechanism to enhance the immune system’s response to vitamin D supplementation. However, more studies are needed in this area.

It has also been suggested that daily doses of 100–200 mcg of selenium consumption, inhibit genetic damage and cancer development in humans (80). About 400 mcg of selenium per day is considered the upper limit. Higher doses of RDA are needed to inhibit genetic damage and cancer. However, it is assumed that taking excessive selenium may cause oxidative damage and lead to genomic instability (81). A study in mice with cancer confirmed the effects of using selenium nanoparticles on cancer-induced cachexia (82). Concomitant use of exercise while improving the immune response, including T cells and antitumor immunity, prevented cachexia in animals (83). It seems that the modulating effects of exercise on selenium-induced responses in cancer can be effective in enhancing antitumor immunity.

The effects of physical exercise on cancer cells have been proven, including activation of invasion and metastasis, escaping growth inhibitors, reducing cell death, inhibiting cancer inflammatory cells, normalizing vessels, escaping immune damage, and reprogramming energy metabolism. Lactic acid is the final metabolite in the anaerobic glycolysis pathway. Increasing aerobic glycolysis in cancer cells can produce a large amount of lactic acid, which reduces pH in cancer cells (84). Proliferation, invasion, and metastasis of cancer cells are associated with angiogenesis, which is related to low pH levels in the tumor microenvironment. Also, lactic acid accumulation in cancer suppresses the immune responses, inhibits the T-cell response, and prevents lactic acid from leaving the T-cell, thereby disrupting the metabolic pathway. It has been stated that moderate-intensity exercise can reduce lactate levels in cells. Therefore, the metabolic process plays a role in the inhibition of anaerobic glycolysis in cancer metabolism (85). As a result, exercise can affect the reprogramming of cancer metabolism by improving the internal blood flow of cancer, angiogenesis, and cancer hypoxia. And rearranging in cancer cells metabolism and taking supplements that are effective in curing cancer, such as selenium and vitamin D, can show a synergistic effect on curing cancer and reducing tumor volume. Using a combination of selenium and other vitamins along with regular physical exercise also seems to be a promising approach to controlling genetic damage, and cancer development. Also, physical exercise can mitigate the side effects of high doses of antioxidants (86).

**FIGURE 1:**
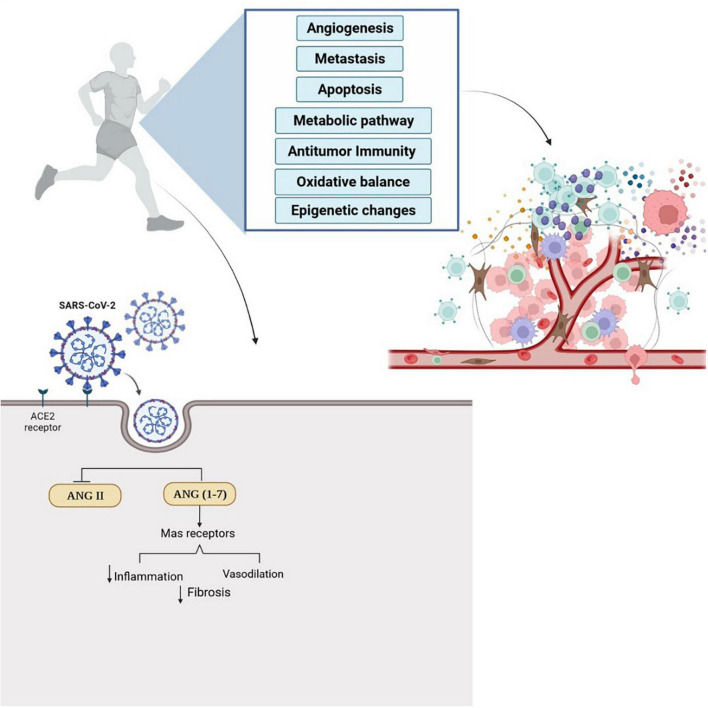
Regular physical exercise can affect tumor microenvironment and induce anti-tumor immunity. Also, exercise training augments the expression of ACE2/ANG-(1-7) Mas receptor and activate anti-inflammatory pathways. Physical exercise simultaneously inhibits the ACE-Ang II-AT1 receptor pathway, inflammatory molecules, and oxidative stress in different tissues and can decrease the possibility of infection with COVID-19. The effects of regular physical exercise on antitumor immunity and ACE2 receptors can probably help cancer patients in the prevention and treatment of COVID-19.

In addition, combining regular physical exercise and dietary supplementation can probably improve innate and acquired immune system responses (48, 87–89) and protect cancer patients against COVID-19. COVID-19 hyper-inflammatory responses and effects on the respiratory system are mediated by the ACE2, which ultimately leads to effects on other organs (90). Two receptors in the renin-angiotensin system by two opposite arms included: one classical composed by ACE/Angiotensin (Ang) II AT1 receptor (AT1R); and the alternative arm comprising ACE2/Ang-(1-7)/Mas receptor that have anti-inflammatory, vasodilatory, antiproliferative, cardioprotective, and renoprotective actions (91, 92). It is shown that COVID-19 is activated with renin-angiotensin system imbalance, which activates the classical arm (ACE/Ang II/AT1R) and leads to pulmonary injury, hematological alterations, and hyper-inflammatory state. Dysfunction of ACE2/Ang-(1-7)/Mas receptor has been observed in some cancers (91). Exercise training can activate the ACE2-Ang1-7-Mas receptor to induce an anti-inflammatory effect; and lead to inhibiting the ACE-Ang II-AT1 receptor pathway, inflammatory molecules, and oxidative stress in different tissues (93). Figure 1 summarizes the possible effects of exercise training in cancer and COVID-19.

In addition, the effects of supplementation in COVID-19 can probably be discussed in more detail concerning the ACE2. Although there is conflicting information about the effects of this supplement on the prevention and treatment of COVID-19, some studies have suggested an active form of Vitamin D improved immune system response against COVID-19 (64). Also, it can probably induce ACE2/Ang-(1-7)/MasR axis activity and inhibits renin and the ACE/Ang II/AT1R axis (94). Vitamin D hydroxylate in the kidney yield 1,25(OH)2D3, and it binds to the VDR to activate vitamin D response elements within target genes (95). Considering the effects of exercise training on VDR, the combination of exercise training and vitamin D supplementation can also be effective in regulating the effects of ACE2 and preventing severe cases of COVID-19 in cancer patients (96). However, more studies are needed, especially in cancer patients with different cancers and at different stages of cancer.

Social distance and vaccination are still the best way to prevent severe cases of COVID-19. Despite the observed effects on immune responses to traditional vaccines, especially in older people (97), there is no information on the possible effects of physical exercise or dietary supplements on the COVID-19 vaccines. More studies with assessment of the combination of exercise training and dietary supplements on the potential efficacy of COVID-19 vaccines may be on the agenda of future studies.

## Conclusion

COVID-19 is spreading with new variants, and cancer patients are prone to severe cases of COVID-19. However, using lifestyle strategies such as regular physical activity and intake of functional compounds as supplements can be effective in treatment outcomes, quality of life, and overall survival. In addition, we cannot ignore the role of regular physical exercise due to metabolic, physiological, and psychological effects. Exercise training and supplementation can improve immune system responses. Combining these two factors can be an important strategy for improving immune responses against COVID-19 and probably improving vaccine responses. However, more studies are needed on nutrition, exercise, and their combined effects on cancer and improving immune responses to infectious diseases in cancer.

## Data Availability Statement

The original contributions presented in the study are included in the article/supplementary material, further inquiries can be directed to the corresponding authors.

## Author Contributions

MM and KS conceived the review, drafted, and approved the final version of the manuscript. AH and AV made some additions to the text, revised the manuscript, and approved the final version. All authors contributed to the article and approved the submitted version.

## Conflict of Interest

The authors declare that the research was conducted in the absence of any commercial or financial relationships that could be construed as a potential conflict of interest.

## Publisher’s Note

All claims expressed in this article are solely those of the authors and do not necessarily represent those of their affiliated organizations, or those of the publisher, the editors and the reviewers. Any product that may be evaluated in this article, or claim that may be made by its manufacturer, is not guaranteed or endorsed by the publisher.
